# Alteration of gray matter texture features over the whole brain in medication-overuse headache using a 3-dimentional texture analysis

**DOI:** 10.1186/s10194-017-0820-4

**Published:** 2017-11-28

**Authors:** Zhiye Chen, Xiaoyan Chen, Zhiqiang Chen, Mengqi Liu, Huiguang He, Lin Ma, Shengyuan Yu

**Affiliations:** 1 Department of Radiology, Chinese PLA General Hospital, Beijing, 100853 China; 2 Department of Radiology, Hainan Branch of Chinese PLA General Hospital, Beijing, 100853 China; 3 Department of Neurology, Chinese PLA General Hospital, Beijing, 100853 China; 4 Research Center for Brain Inspired Intelligence, Institute of Automation, Chinese Academy of Sciences, Beijing, 100190 China; 5 University of Chinese Academy of Sciences, Beijing, 100190 China; 6 Center for Excellence in Brain Science and Intelligence Technology, Beijing, 100190 China

**Keywords:** Magnetic resonance imaging, Medication-overuse headache, Migraine, Texture analysis, Voxel-based gray-level co-occurrence matrix

## Abstract

**Background:**

Imaging studies have provided valuable information in understanding the headache neuromechanism for medication-overuse headache (MOH), and the aim of this study is to investigate altered texture features of MR structural images over the whole brain in MOH using a 3-dimentional texture analysis.

**Methods:**

Brain three-dimensional T1-weighted structural images were obtained from 44 MOH patients and 32 normal controls (NC). The imaging processing included two steps: gray matter (gray images) segment and a 3-dimensional texture features mapping. Voxel-based gray-level co-occurrence matrix (VGLCM) was performed to measure the texture parameters mapping including Contrast, Correlation, Energy, Entropy and inverse difference moment (IDM).

**Results:**

The texture parameters of increased Contrast and Entropy, decreased Energy and IDM were identified in cerebellar vermis of MOH patients compared to NCs. Increased Contrast and decreased Energy were found in left cerebellum. Increased Correlation located in left dorsolateral periaqueductal gray (L-dlPAG), right parahippocampal gyrus (R-PHG), and left middle frontal gyrus (L-MFG) and decreased Correlation located in right superior parietal lobule(R-SPL). Disease duration was positively correlated with Contrast of vermis and negatively correlated with Correlation of R-SPL.HAMD score was negatively correlated with Correlation of R-PHG. MoCA score was positively correlated with Correlation of R-SPL.

**Conclusion:**

The altered textures in gray matter related to pain discrimination and modulation, affective and cognitive processing were helpful in understanding the pathogenesis of MOH. Texture analysis using VGLCM is a sensitive and efficient method to detect subtle gray matter changes in MOH.

**Electronic supplementary material:**

The online version of this article (10.1186/s10194-017-0820-4) contains supplementary material, which is available to authorized users.

## Background

Medication-overuse headache (MOH) is a secondary chronic headache in patients with a pre-existing primary headache disorder caused by regular overuse of acute or symptomatic headache medication for more than 3 months [1]. MOH was one of the most prevalent neurological disorders to cause disability [2]. Medication withdrawal was the primary treatment but only a few patients may achieve improvement [3]. However, the mechanism of MOH generation still remains unclear. Imaging studies have played a role in elucidating the pathophysiological changes of MOH by finding alteration in various aspects of pain processing and reward system [4]. In the published documents, the common MRI technique about headache included conventional T2WI [5], advanced brain structure segment [6–8], resting-state functional MRI [9], and diffusion kurtosis imaging (DKI) [10], which provided much more valuable information to understand the headache neuromechanism. However, this advanced MRI technique may consume much more labour and time, and some functional test might also be influenced by some uncontrolled state such as motion and emotion.

Texture features are the intrinsic properties of images, and reflects the degree of gray distribution, contrast, spatial distribution and other image characteristics [11]. Texture analysis plays a key role in the image analysis, and it could make the invisible intrinsic image characteristics visible [12]. Recent studies presented that texture analysis had widely been applied in the clinical practice, such as rectal cancer [13, 14], hepatic hemangioma [15], mild cognitive impairment [16], glioblastoma [17], and etc. In our previous study [18], we performed a pilot texture feature analysis for periaqueductal gray (PAG) using a 2-dimentionalgray-level co-occurrence matrix (2D–GLCM) and primary results suggested increased Contrast presented in MOH patients. However, 2D–GLCM might be influenced by some factors, such as the size of the step in pixels and the direction of the step, and manual measurement. Therefore, it was limited in the clinical practice. In recent documents [19, 20], 3-dimentional(3D) texture analysis technique were reported, and it had some valuable merits, such as voxel-based analysis over the whole brain, and not influenced by the size and the direction. However, the main limit for the previous study was the calculation time, which would need 15–30 min for one texture map [19]. Therefore, the optimization of the 3D texture calculation would improve the clinical application of this texture technique.

In the current study, we hypothesize that there’s altered texture feature of gray matter in MOH patients. To address this hypothesis, we prospectively acquired high resolution structural images from 44 MOH patients and 32 normal controls (NC). Secondly, voxel-based GLCM optimize and improve the texture calculation efficacy. Lastly, the five texture feature maps were generated and performed with voxel-based analysis over the whole brain to identify the brain regions with abnormal texture changes in MOH.

## Methods

### Subjects

The current study was approved by the local institutional review board, and Written informed consent was obtained from all participants according to the approval of the ethics committee. Forty-four MOH patients were enrolled from the International Headache Center, Department of Neurology, Chinese PLA General Hospital. The inclusion criteria should be fulfilled as following: 1) diagnosis of 8.2 MOH, and 1.1 and 1.2 migraine based on the International Classification of Headache Disorders, third Edition (beta version) (ICHD-III beta) [1]; 2) no migraine preventive medication used in the past 3 months. The exclusion criteria were the following: 1) with any chronic disorders, including hypertension, diabetes mellitus, cardiovascular diseases, cerebrovascular disorders, neoplastic diseases, connective tissue diseases, other subtypes of headache, chronic pain other than headache, severe anxiety or depression preceding the onset of headache, psychiatric diseases, etc.; 2) with alcohol, nicotine, or other substance abuse; 3) with psychotic disorder and regular use of a psychoactive or hormone medication. Thirty-two normal controls (NCs) were recruited from the hospital’s staff and local community. NCs should never have had any primary headache disorders or other types of headache in the past year, and the exclusion criteria was the same with MOH’s exclusion criteria. General demographic and headache information were registered and evaluated in our headache database. All the patients were given with the Visual Analogue Scale (VAS). All the participants were evaluated by the Hamilton Anxiety Scale (HAMA) [21], the Hamilton Depression Scale (HAMD) [22], and the Montreal Cognitive Assessment (MoCA) Beijing Version (http://www.mocatest.org). All imaging protocols were identical for all the subjects. Alcohol, nicotine, caffeine, and other substances were avoided for at least 12 h before MRI examination.

### MRI acquisition

All MRI data were acquired on a GE 3.0 T MR system (DISCOVERY MR750, GE Healthcare, Milwaukee, WI, USA) and a conventional eight-channel quadrature head coil was used. Firstly, conventional T2-weighted imaging (T2WI) and T1 fluid-attenuated inversion recovery (T1-FLAIR) weighted imagingwere acquired to exclude the subjects with obvious structural damage and T2-visible lesion. Secondly, the brain structural images were obtained bya three-dimensional T1-weighted fast spoiled gradient recalled echo (3D T1-FSPGR) sequence generating 180 contiguous axial slices [TR (repetition time) = 6.3 ms, TE (echo time) = 2.8 ms, flip angle = 15o, FOV (field of view) = 25.6 cm × 25.6 cm, Matrix = 256 × 256, NEX (number of acquisition) = 1].

### MR image processing

All the data were processedusing Statistical Parametric Mapping 12 (SPM 12) (http://www.fil.ion.ucl.ac.uk/spm/), FSL (v5.0) (https://fsl.fmrib.ox.ac.uk/fsl/fslwiki/)and MATLAB 7.6 (The Mathworks, Natick, MA, USA). The imaging processing included two steps: gray matter (gray images) segment and a 3-dimensional texture features mapping.

**Fig. 1:**
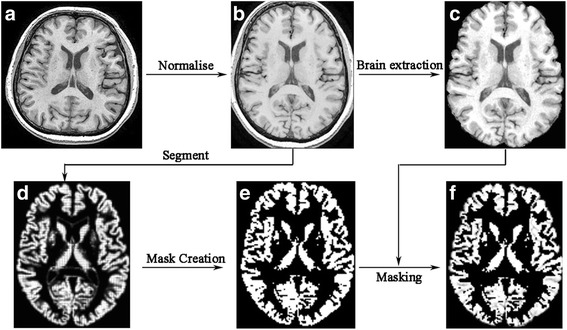
The segments of gray matter in the brain including normalize, brain extraction, mask creation and masking to generate segmented gray matter. (**a**), raw T1 image; (**b**), normalized T1 image; (**c**), normalized T1 images without non-brain tissue; (**d**), gray matter probability images; (**e**), binary gray matter mask; (**f**), segmented gray matter

First step was performed with the following procedures: (1) the raw brain structural images (3D T1-FSPGR) (Fig. 1a) were normalized into MNI space, and the normalized T1 images were obtained (Fig. 1b), which were gray images while not probability images. (2) The normalized T1 images were performed with brain extraction using FSL (v5.0) tool to delete the non-brain tissue, and obtained a good brain T1 images (Fig. 1c). (3) The normalized T1 images were performed with DARTEL segment [23], and gray matter images (tissue probability images) were obtained (Fig. 1d). (4) The gray matter images were performed with binary mask creation, and the gray matter mask was acquired (Fig. 1e). (5) The T1 images without non-brain tissue (Fig. 1c) were masked with the previous gray matter mask, and the final gray matter of the brain (gray images) was obtained (Fig. 1f).

**Fig. 2:**
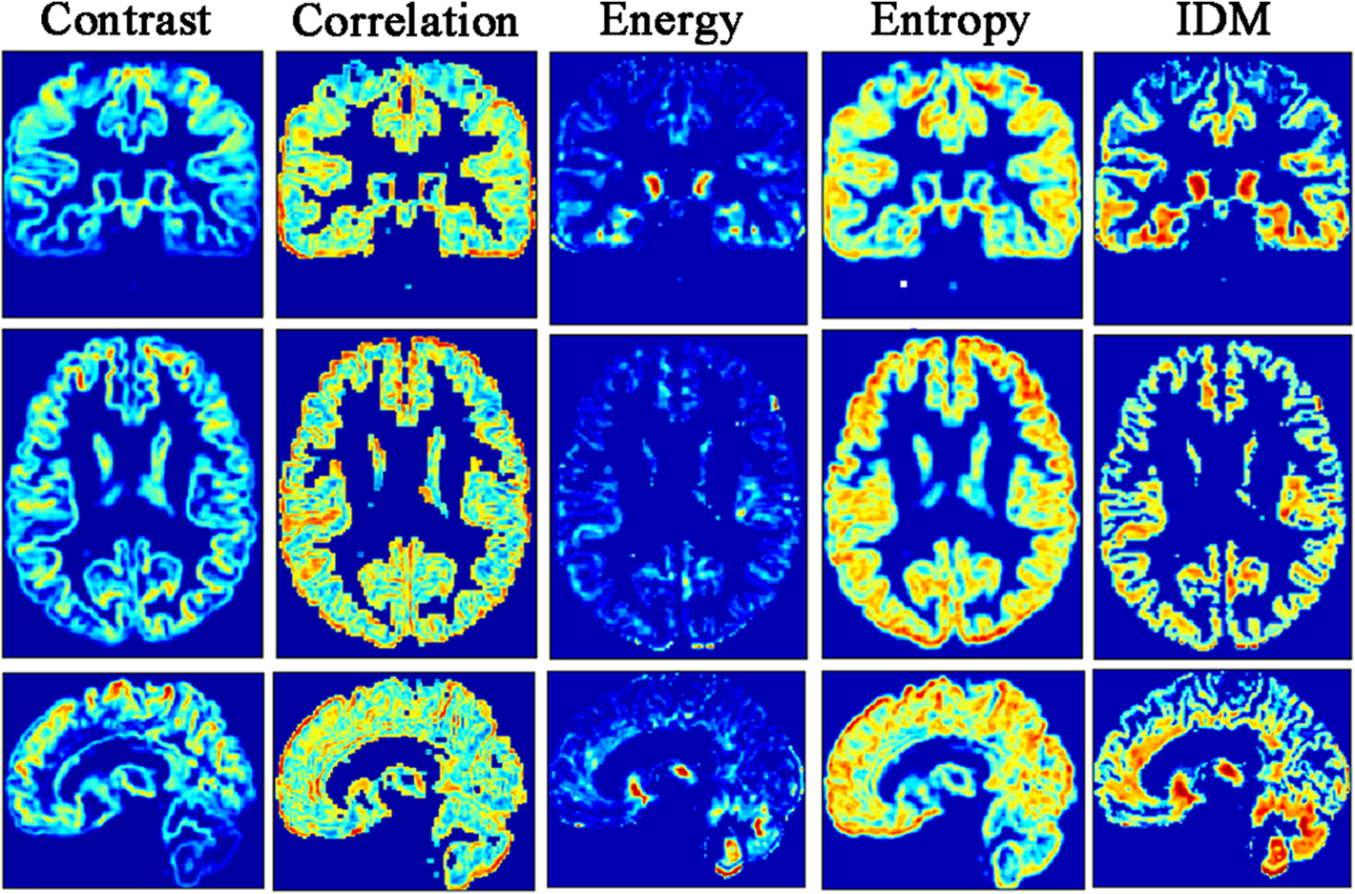
The texture features maps. 1^st^column, Contrast map; 2nd column, Correlation map; 3rd, Energy map; 4th, Entropy map; 5th, IDM map

Following processing was the 3-dimentional texture maps generation. All the texture maps were generated by improved voxel-based gray-level co-occurrence matrix(VGLCM) method [20], and the texture parameters included Contrast, Correlation, Energy (angular second moment, ASM), Entropy and Inverse Difference Moment (IDM) [15, 18]. The texture maps were calculated by an in-house script written on MATLAB (the Math Works, Inc., Natick, MA, USA) platform. The in-house script was provided in Additional file 1. The key settings of the texture maps calculation were the radius R of a spherical region of interest around each voxel and the maximum distance d for the texture points. In the current study, the R was set as 5 mm, and d was set as 1 mm. The calculation time of each brain gray matter was 55 s to generate the five texture features maps (Fig. 2).

The positive clusters were saved as masks to extract the texture values, and then were performed correlation analysis with the clinical variables (including VAS score, disease duration, HAMA score, HAMD score, MoCA score).

### Statistical analysis

The statistical analysis was performed by using PASW Statistics 18.0. The data with normal distribution was described as mean ± standard deviation, and performed with independent two-sample t test and Pearson correlation analysis. The data without normal distribution was described as median (P25, P75), and performed with Mann-Whitney U test and Spearman correlation analysis. The qualitative data (such as sex) were performed with Chi-Square test. Significant difference was set at a *P* value of <0.05.

Voxel-based texture features analysis over the whole brain was performed between MOH and NC, and two-sample t-test design model was selected to identify the brain regions with significant altered texture features in MOH. Age and gender were considered as covariates, and significance was set at a *P* value of <0.001 without correction.

## Results

### Demography and neuropsychological evaluation

Forty-four MOH patients (F/M 36/8) and 32 NC (20/12) were enrolled. Table 1 demonstrated that there was no significant difference for age and sex between MOH and NC. However, significant increased HAMA and HAMD scores and decreased MoCA score were identified in MOH compared with NC (*P* < 0.05).

**Table 1:** The clinical characteristics of MOH patients and normal controls

	MOH	NC	T value	*P* value
Num(F/M)	44(36/8)	32(20/12)	2.64^a^	0.10
Age	42.30 ± 9.62	41.34 ± 10.89	0.40	0.69
HAMA	18.25 ± 8.74	10.00(8.25,13.00) ^b^	4.24^c^	0.00
HAMD	19.80 ± 11.85	8.03 ± 4.34	5.34	0.00
MoCA	23.43 ± 3.72	27.16 ± 2.34	5.00	0.00
DD(yrs)	20.00(10.00,20.00) ^b^	NA	NA	NA
VAS	8.00(7.25,10.00) ^b^	NA	NA	NA

*MOH* medication-overuse headache, *NC* normal control, *DD* disease durationm, *VAS* Visual Analogue Scale, *HAMA* Hamilton Anxiety Scale, *HAMD* Hamilton Depression Scale, *MoCA* Montreal Cognitive Assessment, *NA* not available

^a^Chi-square test

^b^Median (P25, P75)

^c^Mann-Whitney Z value

### Voxel-based comparison of contrast map between MOH and NC

**Fig. 3:**
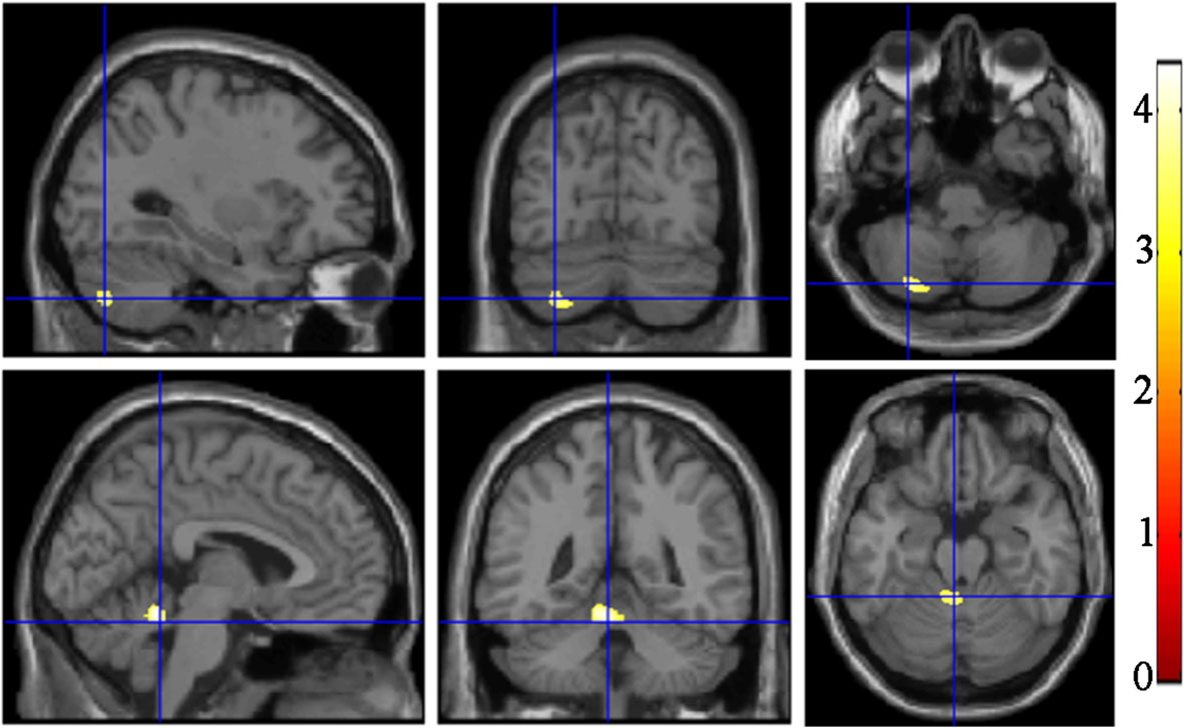
The brain region with increased Contrast in the leftcerebellarCrus and Vermis in MOH compared with NC

Regional increased Contrast located in the right cerebellar Crus and Vermis in MOH compared with NC (Table 2 and Fig. 3). There were no brain regions with decreased Contrast in MOH.

**Table 2:** The brain regions with altered texture features over the whole brain between MOH and NC

Texture		BA	Anatomic region	MNI-space			Cluster size	*P*_uncorr_	Peak T value
				X	Y	Z			
Contrast									
	MOH > NC	NA	Vermis	−3	−47	−18	270	0.000	4.33
		NA	L-cerebellar Crus	−30	−74	−44	158	0.000	3.72
Correlation									
	MOH > NC								
		30	R-PHG	18	−30	−14	41	0.000	4.41
		8	L-MFG	−24	17	45	41	0.000	3.74
		NA	L-dlPAG	−2	−30	−9	61	0.000	3.90
	MOH < NC								
		7	R-SPL	30	−66	39	51	0.000	3.76
Energy									
	MOH < NC		Vermis	0	−47	−21	316	0.000	3.87
			L-Cerebellum Exterior	−11	−45	−12	66	0.000	3.64
Entropy									
	MOH > NC		Vermis	3	−47	−23	438	0.000	4.17
IDM									
	MOH < NC		Vermis	−2	−47	−20	172	0.000	4.37

*L* left hemisphere, *R* right hemisphere, *SPL* superior parietal lobule, *PHG* parahippocampal gyrus, *MFG* middle frontal gyrus, *dlPAG* dorsolateral periaqueductal gray

**Fig. 4:**
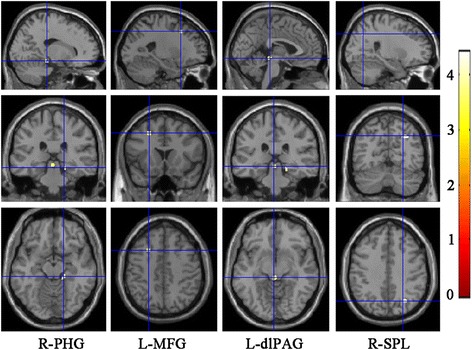
The increased (1–3 column) and decreased Correlation in MOH compared with normal controls. R, right hemisphere; L, left hemisphere; PHG, parahippocampal gyrus; MFG, middle frontal gyrus; dlPAG, dorsolateral periaqueductal gray; SPL, superior parietal lobule

The correlation analysis presented that there was a significant relation with disease duration (*r* = 0.31, *P* value = 0.04) (Table 3 and Fig. 4). The other texture value of positive clusters showed no significant relation with the clinical variables.

**Table 3:** Correlation analysis between positive clusters’ texture value and clinical variables in MOH

Texture	Cluster	Texture value	VAS^b^		DD^b^		HAMA		HAMD		MoCA	
			r value	*P* value	r value	*P* value	r value	*P* value	r value	*P* value	r value	*P* value
Contrast												
Increased	L-Cerebellar Crus	1.11(0.69,1.52) ^a^	−0.06	0.72	−0.07	0.64	−0.16	0.32	−0.14	0.36	0.27	0.08
	Vermis	1.20 ± 0.46	−0.23	0.14	0.31	0.04	−0.06	0.69	−0.10	0.50	−0.09	0.55
Correlation												
Increased	R-PHG	91.36 ± 23.25	−0.12	0.43	0.05	0.76	−0.23	0.13	−0.39	0.01	0.12	0.43
	L-MFG	77.16(70.31,86.29) ^a^	0.22	0.15	0.00	0.98	0.05	0.78	0.15	0.35	0.01	0.96
	L-dlPAG	82.60 ± 7.94	−0.25	0.1	0.19	0.22	−0.11	0.50	−0.13	0.40	−0.01	0.97
Decreased	R-SPL(BA7)	72.58(65.79,78.01) ^a^	0.01	0.93	−0.33	0.03	−0.12	0.44	−0.12	0.44	0.37	0.01
Energy												
Decreased	Vermis	36.86 ± 10.03	−0.12	0.43	0.05	0.76	−0.02	0.89	−0.05	0.74	0.03	0.87
	L-Cerebellum Exterior	32.90 ± 10.21	0.22	0.15	0.00	0.98	−0.02	0.88	−0.12	0.44	0.01	0.95
Entropy												
Increased	Vermis	185.71 ± 33.77	−0.07	0.67	0.22	0.16	−0.06	0.69	−0.03	0.87	0.04	0.82
IDM												
Decreased	Vermis	82.32 ± 5.04	0.14	0.37	0.22	0.14	−0.01	0.97	0.02	0.88	0.10	0.54

*L* left hemisphere, *R* right hemisphere, *SPL* superior parietal lobule

^a^Median (P25, P75) with Spearman correlation analysis

^b^Spearman correlation analysis

### Voxel-based comparison of correlation map between MOH and NC

**Fig. 5:**
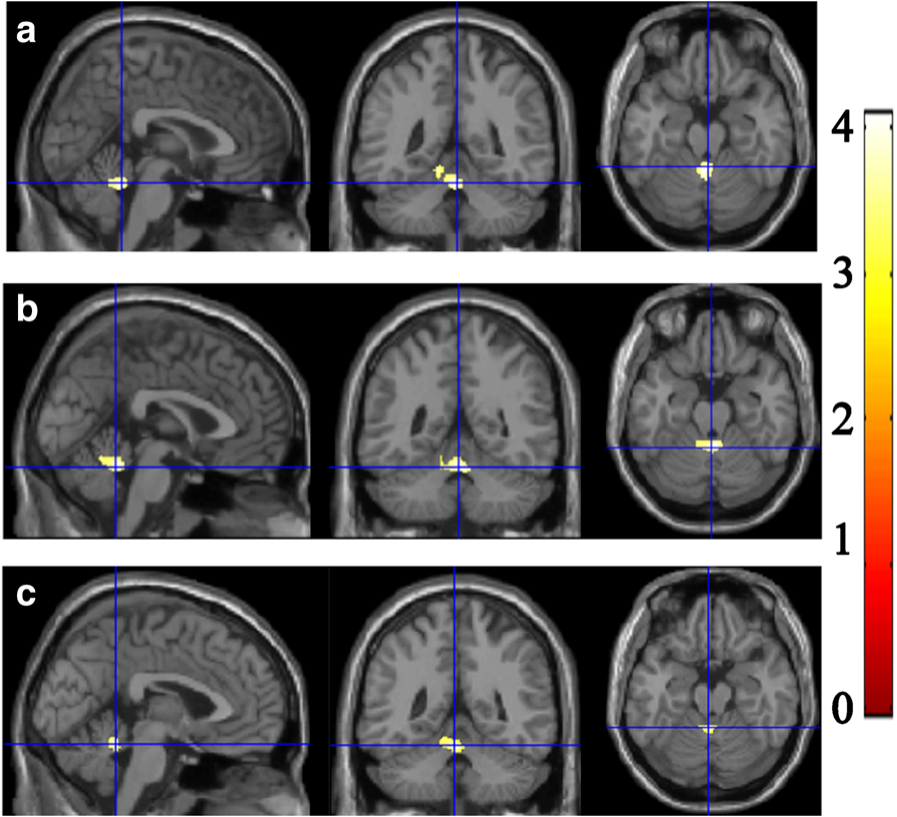
Alteration of Texture Energy, Entropy and IDM. Line (**a**), The brain regions with decreased Energy located in vermis and left cerebellum exterior in MOH; (**b**), Line **b** and **c**, The brain region with increased Entropy and decreased IDM both located in vermis in MOH

Table 2 demonstrated that the brain regions with increased Correlation located in right parahippocampal gyrus (R-PHG), left middle frontal gyrus (L-MFG) and left dorsolateral periaqueductal gray (L-dlPAG) in MOH patients compared with NC. The decreased Correlation located in right superior parietal lobule (Table 2 and Fig. 5).

Figure 4 presented that there was a negative correlation between the Correlation value of R-PHG and HAMD score (*r* = −0.39, *P* = 0.01). Besides, the Correlation value of R-SPL presented negative relation with disease duration (*r* = −0.33, *P* value = 0.03), and positive relation with MoCA score (*r* = 0.37, *P* value = 0.01) (Table 3).

### Voxel-based comparison of energy, entropy and IDM map between MOH and NC

**Fig. 6:**
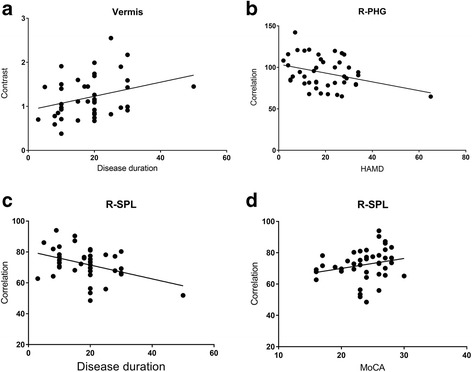
Scatter plot of texture parameters with clinical variables. **a** Increased Contrast of vermis presented significant positive relation with disease duration in MOH; **b** Increased Correlation of right parahippocampal gyrus (R-PHG) presented significant negative relation with HAMD score; **c** Decreased Correlation of right superior parietal lobule (R-SPL) presented significant negative relation with disease duration; **d** Decreased Correlation presented significant positive relation with MoCA score

The vermis presented decreased Energy and IDM, and increased Entropy in MOH compared with NC (Table 2 and Fig. 6). Left cerebellum exterior also presented decreased Energy in MOH.

The correlation analysis demonstrated there was no any correlation between the texture value of above positive clusters with the clinical variable (Table 3).

## Discussion

This is the first study to analyze the brain regions with abnormal texture changes in MOH by using improved voxel-based gray-level co-occurrence matrix (VGLCM) method [20]. Compared with voxel-based morphometry to observe the subtle volume changes, this method could detect the fine changes in tissues image intrinsic characteristics with multiple texture parameters mapping and have higher calculation efficacy [20]. Five texture parameters including Contrast, Correlation, Energy (angular second moment, ASM), Entropy and IDM were extracted in this study [15, 18]. Previous studies recognized that Contrast represented the amount of local gray level variation, Correlation represented the linear dependency of grey levels of neighboring pixels, Energy represented image homogeneity, Entropy represented the amount of information of the image that is needed for the image compression, and IDM represented the local homogeneity [12]. The texture map analyses in the present study revealed altered texture features in cerebellar vermis, left cerebellum, left dorsolateral periaqueductal gray (L-dlPAG), right parahippocampal gyrus (R-PHG), left middle frontal gyrus (L-MFG) and right superior parietal lobule(R-SPL).

The texture parameters of increased Contrast and Entropy, decreased Energy and IDM in cerebellar vermis of MOH patients indicated that the texture feature of vermis in MOH was more heterogeneous and complicated in MR T1 images. The texture feature of left cerebellum was also more heterogeneous in MOH due to increased Contrast and decreased Energy. The cerebellum has been recently recognized to be associated with cognitive, sensorimotor, pain and affective information processing [24].In a 18-FDG PET study, cerebellar vermis was hypermetabolic in MOH patients but recovered to normal 3 weeks after drug withdrawal [25], indicating hypermetabolic status may contribute to increased texture heterogeneity. Patients of familial hemiplegic migraine (FHM) with CACNA1A gene mutation may develop progressive cerebellar signs, and MRI revealed predominant cerebellar vermis atrophy [26] while proton MR spectroscopy (1H–MRS) found reduced NAA, Glu and elevated ml in the superior cerebellar vermis indicating regionally distinct neuronal impairment [27]. The relationship of FHM and cerebellar vermis atrophy and metabolic abnormality indicated that cerebellar vermis might be genetically vulnerable to injury in migraine patients. Besides, VBM studies found altered grey matter volume of cerebellum and vermis in MOH [28, 29] but not in chronic migraine without medication overuse [28], supporting that cerebellum was involved in the pathophysiology of MOH. With the evidence of reversible metabolic abnormality of vermis in MOH [25], the change of cerebellar vermis might better be the result of migraine transformation to MOH. The positive correlation of Contrast in vermis with disease duration further suggested the possibility of secondary change of texture feature in genetically vulnerable vermis as a result of migraine chronification with medication overuse. Longitudinal studies would be required to explore this further.

Correlation map in our study demonstrated that increased Correlation located in left dorsolateral periaqueductal gray (L-dlPAG), right parahippocampal gyrus (R-PHG), and left middle frontal gyrus (L-MFG) and decreased Correlation in right superior parietal lobule(R-SPL). A high correlation texture means high predictability of pixel relationships.PAG is considered as a pivotal center in either generation of migraine or in its regulation [30]. Functional and structural MRI studies demonstrated increased iron deposition [31], decreased functional connectivity [9], increased volume [6, 7]and nonspecific hyperintensity lesions [5] of PAG in migraine and MOH patients. Our previous pilot texture analysis using 2D–GLCM found increased contrast in PAG suggesting increased local gray level variation in MR T1 images [7]. The difference of positive texture parameters in PAG may be influenced by the sensitivity of the two different texture analystic methods. However, both texture analyses found changes of texture feature of PAG and provided evidence of PAG in the pathogenesis of MOH. Like PAG, decreased functional connectivity density of R-PHG was also found in MOH in our previous study [32]. Decreased volume [33] and prolonged T2 relaxation times [34] in R-PHG were identified in smoker and alcohol-use disorders, which suggested R-PHG might participate in the dependence related processing. Gray matter volume reduction of R-PHG was also reported in subthreshold depression [35]. Our study found significantly higher HAMD score in MOH patients and a negative correlation between the Correlation value of R-PHG and HAMD score, which further identified the possible anatomic basis for emotional change and dependence behavior of MOH patients. Altered functional connectivity and grey matter volume of L-MFG [36, 37] and SPL [38, 39] were also found in migraineurs indicating abnormalities in network of pain modulation and discrimination in migraine [39]. A former fMRI study found R-SPL was hypoactive in MOH patients and recovered to almost normal 6 months after drug withdrawal [40], which suggested a modification of the pain network in MOH. In our study, we found that the Correlation value of R-SPL was negatively correlated with disease duration, which further suggested neural plasticity with repetitive pain discrimination in MOH. Furthermore, we found that the Correlation value of R-SPL was positively correlated with MoCA. A recent study using arterial spin-labeled perfusion MRI demonstrated significant positive correlations between cerebral blood flow in SPL and Mini-mental State Examination(MMSE) scores in patients with Alzheimer’s disease [41], which suggested a role of SPL in the cognition network.

The current study using an improved VGLCM method can explore whole-brain pathology and brain-behavioral relationships. However, there are several limitations in our study. Firstly, only five texture features were calculated in this study, and more texture features may be considered to screen the significant texture features for MOH patients in the future. Secondly, only 3D high resolution T1-weighted images were used to calculate the texture features, and the other MR images such as T2 weighted image, diffusion weighted image and susceptibility weighted image may also be considered for the texture analysis in the future. Thirdly, dependence behavior was not evaluated in our study and Severity of Dependence Scale (SDS) for MOH patients should be considered for clinical correlation analyses in the future studies. Lastly, longitudinal studies are needed to better identify the dynamic changes of texture feature in MOH.

## Conclusions

In conclusion, this study revealed altered texture feature of several brain regions in MOH patients, which may reflect the neural plasticity of pain discrimination and modulation, affective and cognitive processing in MOH and were helpful in understanding the pathogenesis of MOH. Texture analysis using improved VGLCM method was sensitive and efficient in detecting subtle structural changes over the whole brain in MOH.

## Additional file

Additional file 1: The voxel-based gray level co-occurrence matrix (VGLCM) was introduced as follows: Gray level co-occurrence matrix (GLCM) is a well-known statistical texture analysis method in 2D gray level image. It also can be extended to define texture features on 3D gray level image. (DOCX 16 kb)

## Abbreviations

dlPAG: Dorsolateral periaqueductal gray
HAMA: Hamilton Anxiety Scale
HAMD: Hamilton Depression Scale
IDM: Inverse difference moment
MFG: Middle frontal gyrus
MoCA: Montreal Cognitive Assessment
MOH: Medication-overuse headache
NC: Normal controls
PHG: Parahippocampal gyrus
SPL: Superior parietal lobule
VAS: Visual analogue scale
VGLCM: Voxel-based gray-level co-occurrence matrix
