# Supplementary material for: Alteration of gray matter texture features over the whole brain in medication-overuse headache using a 3-dimentional texture analysis
Source: J Headache Pain. 2017 Nov 28;18(1):112. doi: 10.1186/s10194-017-0820-4 (PMC5745370; doi:10.1186/s10194-017-0820-4)
Supplement: Additional file 1: — The voxel-based gray level co-occurrence matrix (VGLCM) was introduced as follows: Gray level co-occurrence matrix (GLCM) is a well-known statistical texture analysis method in 2D gray level image. It also can be extended to define texture features on 3D gray level image. (DOCX 16 kb) [file 10194_2017_820_MOESM1_ESM.docx]

Additional file 1 The voxel-based gray level co-occurrence matrix (VGLCM) was introduced as follows:

Gray level co-occurrence matrix (GLCM) is a well-known statistical texture analysis method in 2D gray level image. It also can be extended to define texture features on 3D gray level image.

In 2D images, a grayscale image, I, is a matrix of numbers which represent intensities in the image. Assume that these numbers (intensities or gray levels) range from 1 to N_g_ and the image has a height of N_x_ and a width of N_y_. Then the image I is represented as a function mapping the spatial domain to the gray value:

$$I:L_{x}\times L_{y}\to G$$

Where $L_{x}=\left\{ 1,\ldots,N_{x} \right\}$, $L_{y}=\left\{ 1,\ldots,N_{y} \right\}$ denote spatial domain along x axis and y axis, and $G=\left\{ 1,\ldots,N_{g} \right\}$ the gray values. Because the intensities range of image is usually different from the gray level $N_{g}$, the image need be transformed into the target gray level by

$$I\left( x,y \right)=\mathrm{ceil}\left( \frac{I\left( x,y \right)}{Q_{g}}N_{g} \right)$$

Where $I\left( x,y \right)$ is the intensity value of the pixel in location (x,y), and the ceil is ceil function which returns the closest and not smaller integer value of the given number.

To define GLCM, an offset should be defined first: a pixel $I\left( x,y \right)$ offset O=[a,b] to $I\left( m,n \right)$, then m=x+a, n=y+b. Under the offset O=[a,b], GLCM_O_ can be defined as:

$$\mathrm{GLCM}_{O}(i,j)=\#\{(I\left( k,l \right),I\left( m,n \right))|$$

$$k,m\in L_{x},l,n\in L_{y},m=k+a,n=l+b,I\left( k,l \right)=i,I\left( m,n \right)=j\}$$

In the other words, the GLCM for a specific offset O is an N_g_xN_g_ matrix where the entry (i,j) shows how many times the pair of I(k,l)=i and I(m,n)=j appears. In the same way, under the 3D offset O=[a,b,c], GLCM in 3D image can be defined as:

$$I:L_{x}\times L_{y}\times L_{z}\to G$$

$$\mathrm{GLCM}_{O}(i,j)=\#\{(I\left( k,l,u \right),I\left( m,n,v \right))|$$

$$k,m\in L_{x},l,n\in L_{y},u,v\in L_{z},m=k+a,n=l+b,v=u+c,I\left( k,l,u \right)=i,I\left( m,n,v \right)=j\}$$

Where $L_{z}=\left\{ 1,\ldots,N_{z} \right\}$ denote spatial domain along z axis.

GLCM shows the texture of the whole image, so it doesn’t shows the information for local texture of a specific voxel. To solve the problem, a spherical region of interest of radius R around each voxel is considered. To alleviate the computational expense, a cube region is applied in practical application. Assume that the location of voxel V is $（V_{x},V_{y},V_{z}）$, then the region of interest with the center of V and edge length of 2R+1 is defined as

$$S^{R}\left( V \right)=\{(x,y,z)|x\in\left\{ 1,\ldots,N_{x} \right\},y\in\left\{ 1,\ldots,N_{y} \right\},z\in\left\{ 1,\ldots,N_{z} \right\},\left| x-V_{x} \right|\leq R,\left| y-V_{y} \right|\leq R,\left| z-V_{z} \right|\leq R\}$$

For a specific offset O=[a,b,c], VGLCM is defined as

$$\mathrm{VGLCM}_{V,R,O}\left( i,j \right)=\#\{(I\left( k,l,u \right),I(m,n,v))|$$

$$\left( k,l,u \right),\left( m,n,v \right)\in S^{R},m=k+a,n=l+b,v=u+c,I\left( k,l,u \right)=i,I\left( m,n,v \right)=j\}$$

For an offset set D_d_={[a,b,c]||a|<=d,|b|<=d,|c|<=d}, VGLCM is defined as

$$\mathrm{VGLCM}_{V,R,D_{d}}\left( i,j \right)=\sum_{\forall O\in D_{d}} {VGLCM}_{V,R,O}\left( i,j \right)$$

Normalized it:

$$\mathrm{VGLCM}_{V,R,D_{d}}\left( i,j \right)=\frac{{VGLCM}_{V,R,D_{d}}\left( i,j \right)}{\sum_{i=1}^{N_{g}} \sum_{j=1}^{N_{g}} {VGLCM}_{V,R,D_{d}}\left( i,j \right)}$$

For a offset set D_d_, there are （2d+1）^3^ offsets, and for a region $S^{R}\left( V \right)$, there are （2R+1）^3^ voxels. Thus it needs at least （2d+1）^3^（2R+1）^3^ times operation if no further algorithm is applied, which cost a heavy computational expense. Fortunately, dynamic programming can be applied to alleviate the computational expense, which leads the complexity from O（d^3^R^3^）to O（R^3^）. It also means that we can take the benefit that the computational expense stays the same no matter how d changes.
